# Development of pH-Responsive, Thermosensitive, Antibacterial, and Anticancer CS/PVA/Graphene Blended Hydrogels for Controlled Drug Delivery

**DOI:** 10.3390/gels10030205

**Published:** 2024-03-18

**Authors:** Saira Mansha, Amna Sajjad, Aneeqa Zarbab, Tahmina Afzal, Zakia Kanwal, Muhammad Javaid Iqbal, Mohsin Ali Raza, Sharafat Ali

**Affiliations:** 1 Department of Zoology, Faculty of Life Sciences, Government College University, Faisalabad 38000, Punjab, Pakistananeeqa.20094680520@gcuf.edu.pk (A.Z.); 2 Centre of Excellence in Solid State Physics, University of the Punjab, Quaid-e-Azam Campus, Lahore 54590, Punjab, Pakistan; tahminaaafzal1234@gmail.com (T.A.); javaid.cssp@pu.edu.pk (M.J.I.); 3 Department of Zoology, Lahore College for Women University, Lahore 44444, Punjab, Pakistan; zakia.kanwal@lcwu.edu.pk; 4 Institute of Metallurgy and Materials Engineering, Faculty of Chemical and Materials Engineering, University of the Punjab, Quaid-e-Azam Campus, Lahore 54590, Punjab, Pakistan; mohsin.imme@pu.edu.pk; 5 Department of Built Environment and Energy Technology, Linnæus University, SE-351 95 Växjö, Sweden

**Keywords:** GNS-based hydrogel, methotrexate, hemocompatible, biodegradable, hepatocellular carcinoma

## Abstract

Drug delivery techniques based on polymers have been investigated for their potential to improve drug solubility, reduce systemic side effects, and controlled and targeted administration at infection site. In this study, we developed a co-polymeric hydrogel composed of graphene sheets (GNS), polyvinyl alcohol (PVA), and chitosan (CS) that is loaded with methotrexate (MTX) for in vitro liver cancer treatment. Fourier transform infrared spectroscopy (FTIR) and atomic force microscopy (AFM) was employed to check the structural properties and surface morphology. Moreover, tests were conducted on the cytotoxicity, hemolytic activity, release kinetics, swelling behaviour and degradation of hydrogels. A controlled release of drug from hydrogel in PBS at pH 7.4 was examined using release kinetics. Maximal drug release in six hours was 97.34%. The prepared hydrogels did not encourage the HepG2 growth and were non-hemolytic. The current study highlights the potential of GNS-based hydrogel loaded with MTX as an encouraging therapy for hepatocellular carcinoma. HepG2 cell viability of MTX-loaded CS-PVA-GNS hydrogel was (IC50 5.87 µg/200 mL) in comparison to free MTX (IC50 5.03 µg/200 mL). These outcomes recommend that hydrogels with GNS ensure improved drug delivery in cancer microenvironment while lessening adverse consequences on healthy cells.

## 1. Introduction

Drug administration devices made of polymers are appropriate and more prevalent methods for efficient and controlled release of drug. Polymer based drug delivery mechanisms include hydrogels, rods, wafers, drug-eluting sheets, and nanoparticles. It guarantees improved drug solubility, less systemic side effects, and drug bioavailability to the affected site [1]. Research on stimuli-responsive polymer systems holds great potential, particularly in the field of drug delivery. These polymers can be designed to release drugs in a targeted and regulated way, leading to less adverse effects and a more successful course of therapy. For example, drugs can be released selectively into regions such as malignant tumors, when pH or temperature changes due to the response of polymers to these changes. Moreover, these polymers have the potential to offer prolonged sustained drug release [2,3].

When polymers are chemically crosslinked with an aqueous solution, hydrogels are formed. Hydrogels, made up of polymers, are becoming more popular as a drug carrier to deliver drugs to particular location, in addition to its use in tissue engineering, regeneration, and biomedical sciences [4].

Hydrogels are available in a wide variety of compositions and have a wide range of biological and physicochemical characteristics. Synthesized hydrogels resemble native extracellular matrix (ECM) in a number of ways. Because of this, synthetic hydrogels have uses in the development of MCS (for drug screening and cancer research). These constraints could be removed by using certain biodegradable cross-linkers and cell adhesion ligands attached on the synthetic hydrogels [5]. An injectable hydrogel that is thermosensitive should be in the solution state at room temperature or below, which makes it simple to load medicines and medicinal proteins, enzymes, or live cells. Raising the temperature to body temperature allows the solution transition into a non-flowing hydrogel, trapping the cargo, which may subsequently be released at the intended location [6]. When compared to other drug delivery systems (DDs), injectable hydrogels provide a number of benefits [7]. Injectable hydrogels might provide high drug loading of anticancer medicines due to their biocompatibility, ease of manufacturing, and inject ability into tumor locations [8]. Another example of a “smart” system—which has been widely used in cancer chemotherapy—are the pH-sensitive DDSs. It is evident that different cellular sections and tissues have significantly varying pH values [9,10]. For example, the pH of the tumor environment is lower than that of normal tissues (pH 7.4) because of the high rate of glycolysis production, but the endosomal pH may vary between 4.5 and 6.5. Due to this pH change, many pH-sensitive DDSs have been developed, which have the potential to target the tumor pH condition, react to pH indicators, and enhance the effectiveness of drug delivery [11]. Chitosan-based hydrogels have attracted a lot of interest in recent years for a number of hydrogel applications due to their exceptional features [12]. Chitosan (CS), a cationic polysaccharide derived from chitin and produced via alkaline deacetylation process, has been widely employed as a biopolymer in the form of scaffold for tissue engineering, membranes, and fibers, as well as gels, for targeted drug administration [13]. It has a number of properties, including biodegradability, biocompatibility, nontoxicity and may be employed in a number of applications, including biomedicine and DDs [14]. It has also been used for the control and treatment of different kinds of cancers i.e., lung cancer, ovarian cancer, RIF-1 fibrosarcoma, breast cancer, cervical cancer, and the cancers that are linked with mucin production [15]. According to Kweon et al. (2003) it has an excellent ability for holding water as well as bioactivity, fat binding capacity, antibacterial activity, and antifungal activity [16]. Furthermore, CS may help drugs penetrate epithelial cells by loosening the tight connections between them [17]. Due to these characteristics, CS is an excellent candidate for developing novel biomedical materials.

Poly vinyl alcohol (PVA) has widely been employed in pharmaceutical and biological applications because of its non-toxicity and excellent biodegradability. It is found to be successful in site-specific delivery of drugs [18].

When graphene is employed as a drug carrier, it is generally converted into an oxidized state (graphene oxide) to improve its hydrophilicity and lower its thickness [19]. Graphene oxide (GO) has an enormous surface area, adsorption capacity, mechanical strength, biocompatibility, colloidal stability. It has pushed cancer nanotechnology to overcome hurdles in cancer therapy, such as integrating anticancer medicines into a three-dimensional hydrogel network using biocompatible polymers as nanocomposite carriers and controlling anticancer drug release. The GO surface, in particular, allows hydrophilic interactions and π-π stacking with anticancer drugs [20]. According to Feng et al. (2022), graphene oxide (GO) has a very large specific surface area, biocompatibility, hydrophilicity, and a varied range of functional groups, including epoxy, carboxyl, and hydroxyl groups [21]. For efficient drug delivery applications, GO needs to be drug-loaded due to its confined dispersion in a physiological media. In order to improve the dispersion of GO, several physiologically active compounds (such as CS) have been employed [22]. Drug distribution and transportation occur through interaction between drug groups and GO functional groups, which is made possible by GO’s enormous surface area. Because of their pH-sensitive drug release behaviour, GO-based hydrogels are known for their relevance in drug release [23]. As a result, GO-based drug delivery systems have been the subject of much study. Controlled drug release was accomplished by varying the pH of the GO-based hydrogel [24]. Despite the recent utilization of GO in hydrogels and some promising results, GO brings some risks to medical applications such as toxicity for healthy cells because it can transfer its acidity to hydrogels which comes from its synthesis via Hummers’ method. The acidic nature of suspensions of GO can deteriorate properties of hydrogel and can also be threatening for health cells. To overcome this challenge of GO, we have decided to us graphene nanosheets (GNS) in this work. GNS used in this work were synthesized via chemical vapor deposition method. These had thicknesses of 1–5 nm and their suspensions in water was neutral (pH 7). Furthermore, these GNS had very few functional groups unlike GO which had enormous functional groups.

Methotrexate (MTX) has chemotherapeutic properties that target cancer cells with overexpressed folate receptors. MTX competes with folic acid and damages the DNA, as observed in several cancer cell lines [25]. Its limited water solubility and adverse effects limit its use in the clinic. As a result, researchers created an MTX-loaded hydrogel to increase solubility, reduce side effects, and treat cancer [26].

In the present study, we aimed to develop a biocompatible and biodegradable bipolymeric hydrogel that is designed to develop MTX on-site DDS by keeping in mind the chemotherapeutic unwanted side effects. Hydrogel based nano-devices can reduce the load of expensive chemotherapeutic drugs and thus offer cost effective treatments. As the hydrogels used in this study are biodegradable, they can be easily eliminated from the body in a short course of time and their pH- responsive property makes them suitable for the treatment of cancer cells keeping in view their acidic microenvironment. In this study MTX-loaded hydrogels were synthesized by employing chitosan, polyvinyl alcohol and GNS with different concentrations of Tetraethoxysilane (TEOS) used as a cross-linker. Synthesized hydrogels are intended to inhibit cancerous cells from proliferating by releasing MTX at a specified location. The medicine will only control cancer by inducing cancer cells to undergo apoptosis, after which the hydrogel will disintegrate without harming any healthy cells.

## 2. Results and Discussion

### 2.1. FTIR Analysis

**Figure 1:**
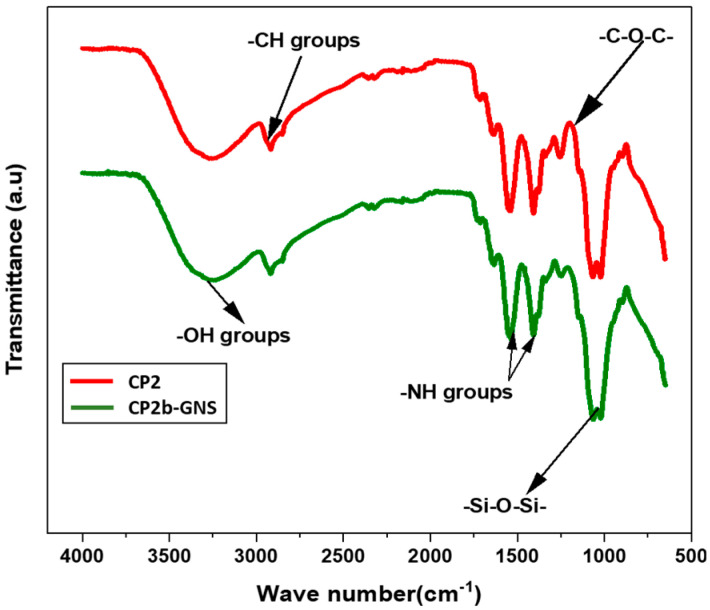
FTIR spectra of the prepared hydrogels indicating the presence of Si-O-Si linkage among OH and NH groups.

A broad band between 3000 and 3500 cm^−1^ shows -OH stretching of intermolecular and intramolecular hydrogen bonding (Figure 1). The characteristics peaks at 2920 and 1246 cm^−1^ show -CH and -C-O-C- stretching, respectively [27]. The peaks at 1400 and 1500 cm^−1^ are attributed to -NH stretching. A characteristic peak of siloxane (-Si-O-Si-) can also be seen at 1065 cm^−1^. The presence of siloxane linkage among chitosan, PVA and GNS in CP2b-GNS is evident from the FTIR investigation [28].

### 2.2. Atomic Force Microscopy

**Figure 2:**
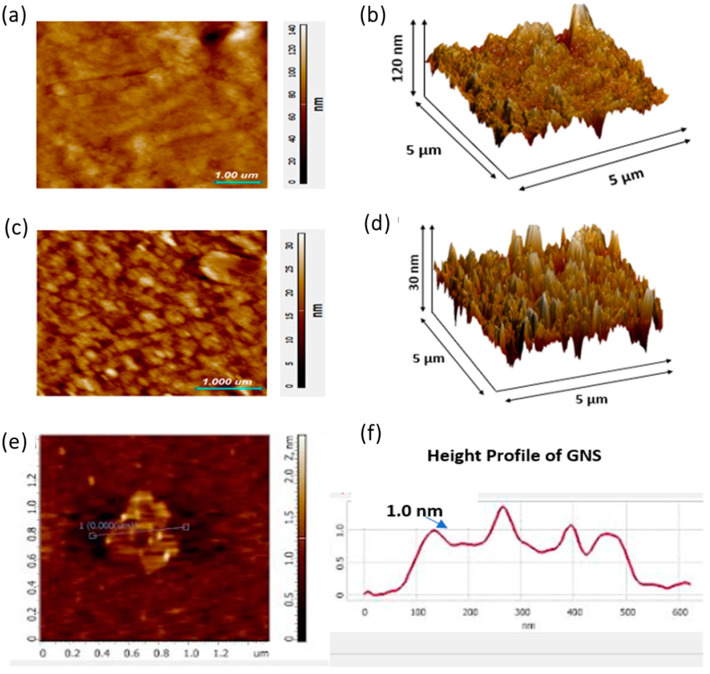
AFM images of the prepared hydrogels (**a**,**b**) CP2b and (**c**,**d**) CP2b-GNS (**e**) GNS (**f**) height profile of GNS showing 1 nm thickness.

AFM was used to study the surface roughness of the prepared hydrogels CP2b and CP2b-GNS. Figure 2 shows the AFM images for both the hydrogel samples and GNS. The measured surface roughness is illustrated in Table 1. It can be seen that CP2b has higher surface roughness than CP2b-GNS. Although CP2b contains the optimized concentration of TEOS cross-linker, it is still left with some porous structure. When GNS is added in cross-linked CP2b, an increased area of GNS shelters those pores [29]. The increased surface area coverage of GNS to the hydrogel with TEOS results in the smooth surface morphology and hence the decreased roughness as compared to CP2b. GNS sheets inherent smoothness also contributed to smoother surface of the hydrogels as can be seen in Figure 2e,f.

### 2.3. Wetting Analysis

**Figure 3:**
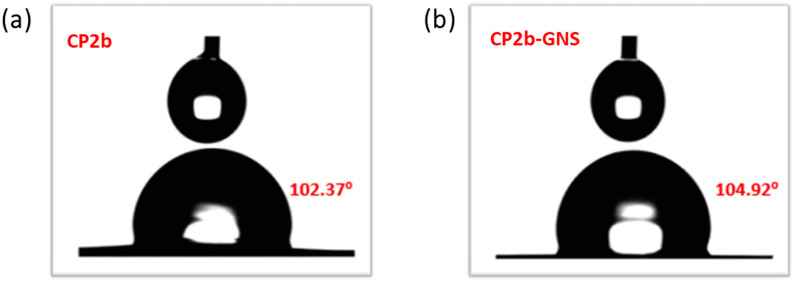
Wetting analysis of the prepared hydrogels (**a**) CP2b and (**b**) CP2b-GNS.

For hydrogel samples, wetting analysis plays an important role in determining whether the hydrogel is hydrophobic or hydrophilic. In this regard, the optimized cross-linking of TEOS is very important as it can shift the behaviour from hydrophilic to hydrophobic [30]. The contact angle of the CP2b is 102.37° which is slightly less than the contact angle of CP2b-GNS (Figure 3). The hydrophilic nature of GO is due to the functional groups available on its surface and edges [28]. In the present work, GNS were synthesized by CVD and have no or very few functional groups available for hydrogen bonding, thus these have hydrophobic nature. Due to their hydrophobic nature, increase in the contact angle of CP2b-GNS is obvious [31]. It can be concluded that CP2b-GNS gel is more hydrophobic than pristine gel.

### 2.4. Swelling Studies

**Figure 4:**
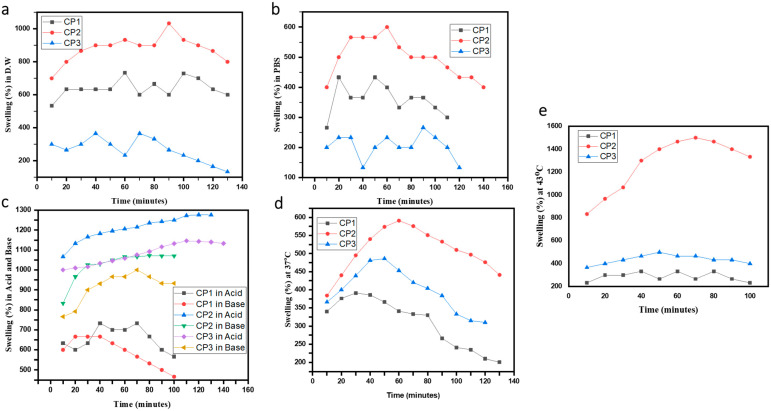
Time vs. Swelling % of CP1, CP2 and CP3 in (**a**) distilled water (**b**) PBS (**c**) acidic (HCl at pH 4) and basic (NaOH at pH 10) solutions (**d**,**e**) effect of temperature of the medium.

The hydrophilic nature of the polymers employed in hydrogels causes them to swell. The water uptake by hydrogels may be several folds of their own weight. The swelling behavior of hydrogels is affected by the medium employed, such as distilled water, acids, and bases. Swelling behavior is critical to understanding the DDS as drug distribution depends on the degree of water absorption by hydrogels; the more the water absorption, the greater the drug diffusion [32]. We investigated the swelling ability of hydrogels prepared with and without TEOS. There was an apparent distinction in the swelling behaviour of cross-linked hydrogels and uncrosslinked (hydrogels without TEOS). In the uncrosslinked hydrogels, the presence of imine linkage was suggested as the potential cause of the maximal degree of swelling. In a hydrogel containing a crosslinker, the imine group may form protons at low pH levels, increasing the intermolecular hydrogen bonding between the protonated imine group and the water molecules [33] resulting in higher swelling (Figure 4c). The swelling ratio is influenced by the pH of the swelling media and is related to the amino groups in the hydrogel network structure. An acidic environment causes hydrogel to swell more. The considerable swelling under acidic environments is due to the charged amino groups (NH_3_^+^) in the polymeric structure, which generates electrostatic repulsion between the polymer chains, permitting maximal water absorption in the network structure [34]. The process of heating tumor cells produces direct cytotoxicity by eliminating cancer cells without harming healthy normal body cells [35]. Therefore, swellings analyses were performed at body temperature (37 °C) and at 43 °C (Figure 4d,e), the recommended temperature for chemotherapy [36].

**Figure 5:**
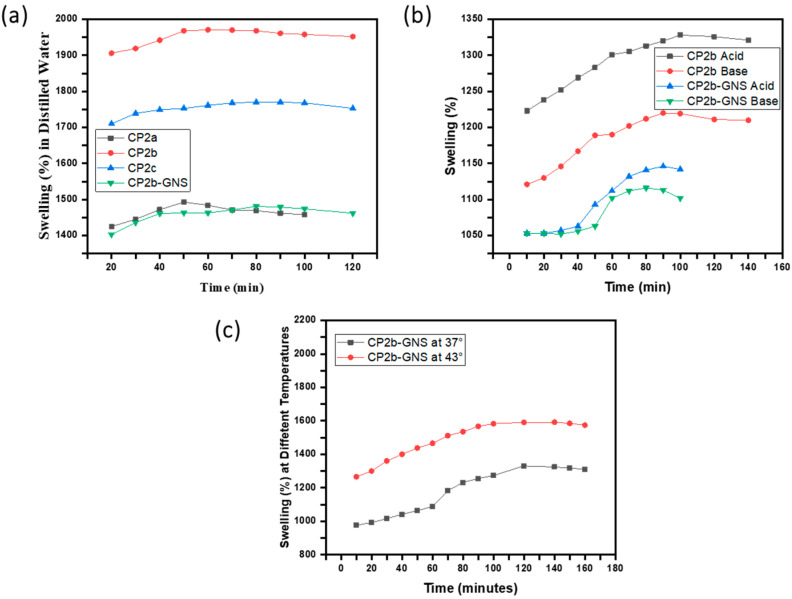
Time vs. Swelling % of CP2a, CP2b, CP3c, and CP2b-GNS in (**a**) distilled water, (**b**) acid-base (HCl at pH 4 and NaOH at pH 10), and (**c**) at different temperatures.

The crosslinked hydrogel CP2b hydrogel gave maximum swelling property (Figure 5). It was observed that by increasing and decreasing the concentration of TEOS from 100 µL the swelling was reduced, which indicates the optimum concentration of TEOS for maximum swelling [32]. The GNS based hydrogel showed lower swelling in distilled water and in acidic and basic solutions (Figure 5). Previously, our co-authors [23] reported that GO incorporation in the same CP2b hydrogel resulted in about 1800% swelling in PBS solution. In this work, CP2b-GNS hydrogel showed lower swelling % in distilled water and acidic and basic solutions, which is mainly attributed to the hydrophobic nature of GNS due to lack of functional groups on GNS.

The higher swelling of hydrogels (CP2b and CP2-GNS) in acidic (at pH 4) and basic (at pH 10) solutions, and at various temperatures (43° and 37°) make them pH sensitive and thermo-responsive. In an alkaline or neutral medium, the amine functional groups deprotonate, converting the NH_3_^+^ groups back to NH_2_. Because of the amino groups in CS, these hydrogels are pH-sensitive [23]. CP2b-GNS has shown a positive temperature response with an increase in swelling by increasing the temperature. The maximum swelling at a high temperature (43 °C) ensures that the prepared hydrogel CP2b-GNS is a suitable candidate for delivering the drug at cancer sites.

### 2.5. Degradation Analysis of Hydrogels

**Figure 6:**
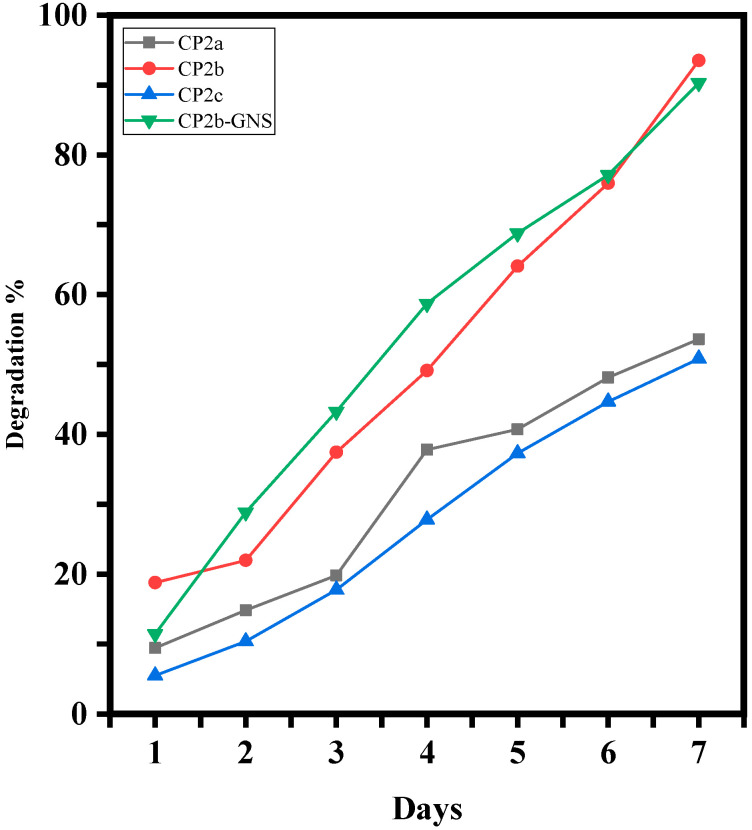
Degradation of CP2a, CP2b, CP2c and CP2b-GNS in PBS media for 7 days.

To determine how well-dried hydrogel films degrade, the degradation of hydrogel films in PBS medium was investigated (Figure 6). The hydrogel sample CP2b was found to disintegrate more quickly than the hydrogel samples CP2a and CP2c. In comparison to CP2a, CP2b, and CP2c, the hydrogel sample CP2b-GNS containing 0.5% of GNS showed much faster degradation. This typical behaviour of hydrogel shows that GNS has not developed bonding with the hydrogel matrix. Previously, our co-authors reported [23] that similar GO-based hydrogel disintegrates slowly in PBS solution which was mainly due to GO ability to form crosslinks with the polymeric matrix of the hydrogel through its functional groups. The comparison confirms that graphene-based hydrogels would have faster degradation.

### 2.6. Hemolysis

**Figure 7:**
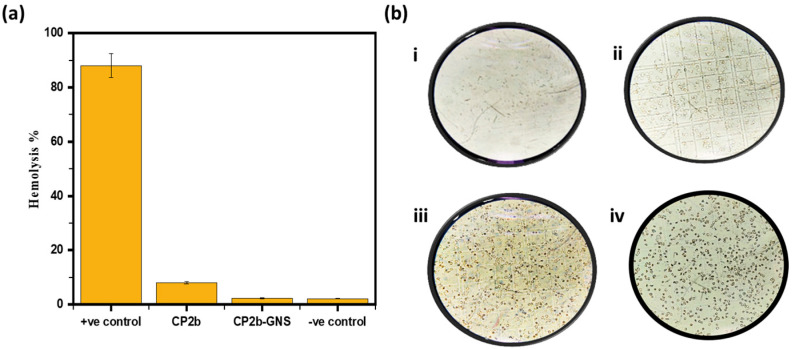
(**a**) Hemolysis % of prepared hydrogels (**b**) Microscopic images of (**i**) +ve Control, (**ii**) CP2b, (**iii**) CP2b-GNS, (**iv**) −ve Control.

To determine if hydrogel was compatible with mammalian cells, hemolytic analysis was carried out. After hydrogel was exposed to erythrocytes from humans for hemocompatibility investigation, the hemolysis percentage to the very toxic surfactant triton-X was determined [32]. In contrast to CP2b, hydrogel CP2b-GNS remarkably showed very little hemolysis (Figure 7a). This demonstrated the hemocompatibility of hydrogel towards human red blood cells. In order to enhance understanding, treated erythrocytes were examined under a microscope. The treated cells had the normal round shape of healthy blood cells, but no cells were seen under a microscope and no survival was shown in the positive control (TX-100) as can be seen in Figure 7b.

### 2.7. Antimicrobial Activity

**Figure 8:**
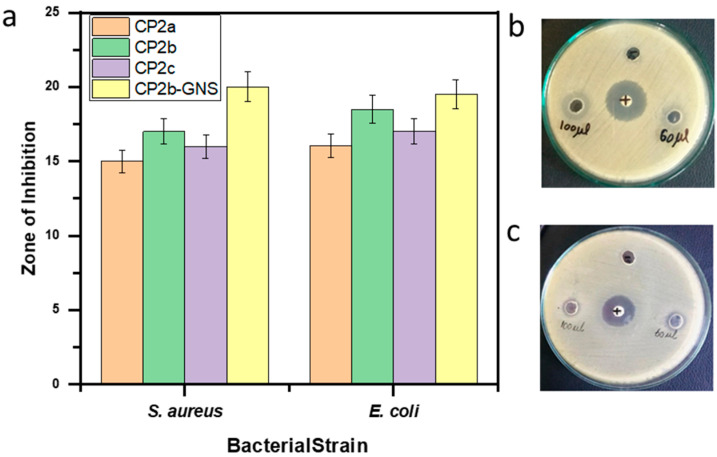
(**a**) Antibacterial activity of CP2a, CP2b, CP2c and CP2GNS against Gram +ve *Staphylococcus aureus* (*S. aureus*) and Gram −ve *Escherichia coli* (*E. coli*), (**b**) Zone of inhibition of CP2b-GNS against Gram +ve *Staphylococcus aureus* (*S. aureus*), (**c**) Zone of inhibition of CP2b-GNS against Gram −ve *Escherichia coli* (*E. coli*).

To examine the antibacterial properties of CP2a, CP2b, CP2c, and CP2b-GNS hydrogels, the well diffusion method was employed. The bacterial strains *S. aureus* and *E. coli* were treated with the hydrogel samples. As shown in Figure 8, each hydrogel sample exhibited antibacterial activity against *S. aureus* and *E. coli*. The increasing concentration of CS in the hydrogels improves antibacterial activity. Following a 24 h incubation period, the zones of inhibition were measured. Clear inhibition zones were observed for all the hydrogel samples. As indicated, CP2b-GNS showed a maximum diameter of 17 and 15.5 mm of the inhibition zone against *S. aureus* and *E. coli*, respectively, while CP2b showed 13 mm and 14.5 mm inhibition zone against *S. aureus* and *E. coli*, respectively (Figure 8). Hydrogels with or without GNS exhibited fair antibacterial activity against the pathogens. In the hydrogel, CS is mainly responsible for the antibacterial characteristic of hydrogel. The ability of CS to readily form connections with bacterial DNA through its NH_2_ gives it an edge over other polysaccharides. This explains why there are greater antibacterial activities as CS concentration rises. Hydrogel regulates the entire bacterium and prevents it from growing further by creating new links with its DNA. Because of their acute edges, GNS ruptures the functional groups and bacterial membranes, controlling the activities of the bacteria [37]. This might contribute to slightly better antibacterial activity of GNS-based hydrogel.

### 2.8. In Vitro Drug Release Analysis

**Figure 9:**
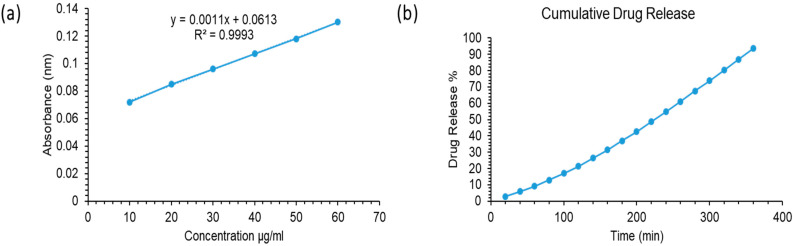
(**a**) Standard Curve of MTX at 321 nm (**b**) Cumulative drug release % of MTX from CP2b-GNS hydrogel.

The findings of a study on the release of the loaded drugs over time in blended hydrogel samples in (PBS) are shown in Figure 9. From the drug-loaded samples, the drug’s release behavior in PBS is linear. The first two hours saw a release of about 18.25% MTX in PBS. MTX has been delivered over an extended period of time; however, after 6 h, it becomes consistent (97.34%).

#### Drug Release Kinetics

In PBS medium (pH 7.4 at 37 °C), the drug release was assessed. The drug release kinetics were assessed using the mathematical models (Equations (1)–(4)).

Zero-order:

(1) Mt=Mo+Kot

First-order:

(2) $$logCo-\frac{kt}{2.303}$$

Higuchi model:

(3) $$ft=Q=KH\times t\frac{1}{2}$$

Korsmeyer–Peppas model:

(4) $$In\frac{Mt}{Mo}=nInt+InK$$

**Figure 10:**
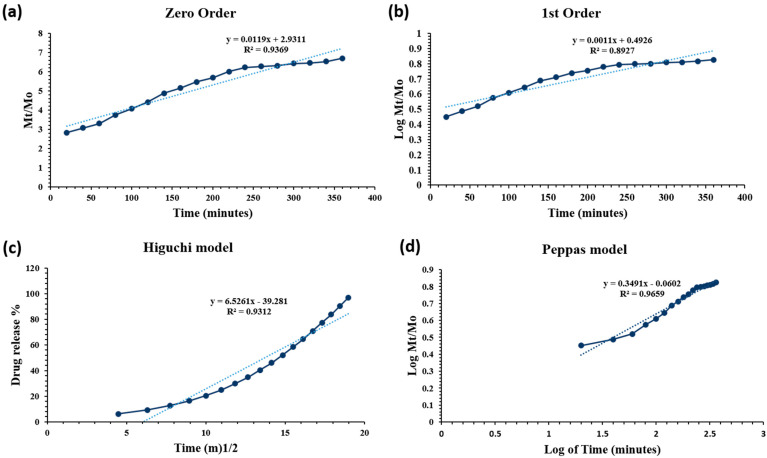
Release kinetics of drug loaded prepared hydrogel, (**a**) zero-order, (**b**) first-order, (**c**) Higuchi model and (**d**) Peppas model.

Drug release is regulated and maintained by the hydrogel’s polymeric matrix. Drug release kinetics are affected by degradation and swelling of hydrogels. The data of drug release was fitted to models: zero and first order, Higuchi, Baker–Lonsdale, Hixson, and Peppas model (Figure 10).

The MTX-loaded CP2b-GNS hydrogel had various kinetic release behaviors, and Table 2 regression coefficient (R^2^) is provided together with other data. The drug release of MTX-loaded hydrogel, follows the Peppas model (also known as Power law model) as it showed the highest value (close to 1) of the regression coefficient (R^2^ = 0.9659). The power-law explains how the polymeric matrix swells and dissolves under the influence of water diffusion [38]. The value of *n* = 0.3491 in Equation (4) corresponds to the drug release mechanism. The n value is close to 0.5 which suggests the drug release is controlled by diffusion of the drug through the matrix.

### 2.9. Cell Viability Assay

**Figure 11:**
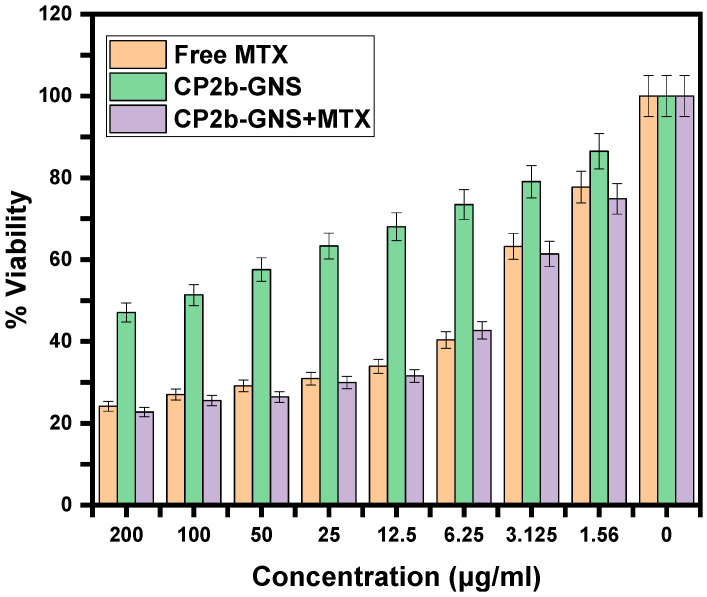
Cytotoxic potential of Free MTX, CP2b-GNS, and CP2b-GNS + MTX against HepG2 cell line at different concentrations after 48 h of treatment.

HepG2 cells were cultured in a medium that contains different concentrations of free MTX, CP2b-GNS and CP2b-GNS+MTX to evaluate therapeutic effectiveness of the hydrogel. The methyl thiazole tetrazolium test was then used to determine the relative cell viability [39]. There is no noticeable toxicity (>80% cell viability) assessed for pure CP2b-GNS at a high concentration of 100 mg/mL for HepG2, showing that the carrier alone is not cytotoxic and the higher efficacy should be expected by the contribution of the drug bound to CP2b-GNS (Figure 11). The water-soluble CP2b-GNS+MTX complexes exerted a greater substantial cytotoxic impact on tumor cells. The 50 percent inhibition of growth was shown by CP2b-GNS+MTX at a concentration (IC50) of ≈5.87 μg/200 mL, whereas free MTX showed 50 percent growth inhibition at a concentration (IC50) of ≈5.03 μg/200 mL. In other words, CP2b-GNS hydrogel can enhance drug effectiveness without increasing the amount of the drug. Because MTX molecules passively diffuse through the plasma membrane, a large dosage is required to achieve this. The endocytosis mechanism by cells accomplishes the internalization of drugs and GNS complexes. Through electrostatic interaction with the negatively charged cell membrane, the positive charges of CS may also enhance cellular absorption of CP2b-GNS+MTX. Furthermore, it is possible that the associated MTX is protected from drug degradation by intracellular enzymes and has a strong affinity for serum albumin, preserving its anticancer action.

## 3. Conclusions

Developing drug delivery systems that facilitate the regulated and spatial distribution of anticancer medications at the tumor site becomes attractive because it may reduce the unfavorable side effects associated with traditional chemotherapy. This suggestion has led to the development of novel hydrogel compositions possessing thermo-responsive characteristics. A new hydrogel that is responsive to temperature and pH was effectively designed by mixing GNS, PVA and CS at varying concentrations of TEOS.

The presence of integrated functional groups and links between the hydrogel’s component parts was validated by FTIR analysis. In contrast, swelling at different pH values suggested that hydrogels swelled more at acidic pH values and less at basic pH values. The swelling tests conducted in water demonstrated a decrease in swelling with an increase in the quantity of crosslinker. Studies on the swelling of hydrogels at several temperatures reveal that the maximum swelling occurred at 43 °C rather than 37 °C. The results of the surface morphology verified the existence of porous structures. The hydrogel samples exhibited bacteriostatic activity against both *E. coli* and *S. aureus*; however, the CP2b and CP2b-GNS hydrogels had the highest levels of activity. The MTX drug release profile indicated that the created formulation may be taken into consideration for the drug’s release. The MTT experiment demonstrated drug-loaded hydrogel’s inhibitory ability. The MTT experiment revealed that the cross-linker’s binding capability determines the synthetic hydrogels’ antiproliferative efficacy. In the given concentration, CP2b-GNS had demonstrated the lowest percentage of viable cells. After comprehensive data analysis, CP2b-GNS emerges as the most authorized hydrogel for on-site drug delivery to target cancer cells among all manufactured hydrogels. The biomedical materials research community will be very interested in CP2b-GNS for both clinical uses and future development.

## 4. Materials and Methods

### 4.1. Materials

Chitosan, Tetraethoxysilane (TEOS) and Polyvinyl alcohol were purchased from were purchased from Merck, Darmstadt, Germany. Graphene oxide nanosheets (GNS), Acetic acid, Ethanol, Phosphate buffer saline (PBS), Hydrochloric acid (HCl), Sodium Hydroxide (NaOH), Dimethyl sulfoxide (DMSO), Nutrient agar and (3-(4,5-Dimethylthiazol-2-yl)-2,5-Diphenyltetrazolium Bromide)) (MTT) were purchased from Sigma-Aldrich, Burlington, MA, USA. HepG2 cell-line (ATCC HB-8065) (Manassas, VA, USA). Distilled water and Methotrexate was obtain from local scientific store and pharmaceutical company.

### 4.2. Methods

#### 4.2.1. Preparation of CS/PVA Hydrogels

Different concentrations of chitosan were dispersed in 1% acetic acid and 20 mL water was added. To obtain a homogeneous and transparent solution, allow it to stir magnetically for 2 h at room temperature. In another flask (0.8 g, 0.6 g and 0.4 g) PVA was added to distilled water at 80 °C with continuous magnetic stirring. Following that, by taking 80:20 (*w*/*w*) ratios of chitosan and polyvinyl alcohol respectively, two solutions were mixed together and were allowed to stir together for another 1 h. After that various amounts of cross linker Tetraethyl Orthosilicate (TEOS) were added and the hydrogel stirred for the next 3 h at 50 °C (Table 3). After this, hydrogel has been stored at room temperature [23].

#### 4.2.2. Preparation of CS/PVA/GNS Hydrogels

**Figure 12:**
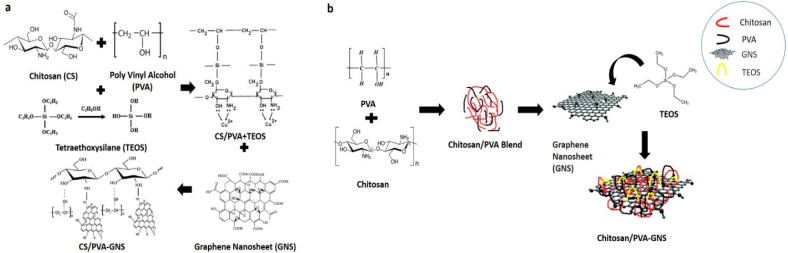
(**a**) Schematic illustration of the expected chemical bonding between chitosan, polyvinyl alcohol and GNS, (**b**) Possible scheme of interaction among CS, PVA, TEOS and GNS.

Ultra-sonication was used to disperse 0.5 percent of GNS in ethanol until it was fully dispersed. The GNS dispersions were then slowly added to the above-mentioned combined solution and aggressively agitated for 1 h [40]. After that TEOS was added and the hydrogel stirred for the next 3 h at 50 °C. After that, hydrogel has been stored at room temperature (Figure 12, Table 3). No other concentration of GNS was used except 0.5% as per the findings published by Khan et al., 2021 [23] (Table 4).

### 4.3. Characterizations

#### 4.3.1. FTIR Analysis

Fourier Transform infrared (FTIR) spectroscopy, in combination with photo acoustic sampling cells and smart ATR accessory, was used for the structural characteristics of produced hydrogels. The spectra were captured in the range of 4000–500 cm^−1^ wavelength with 256 scans as an average at 8 cm^−1^ resolution (USA) by utilizing a Thermo Nicolet 6700 P FTIR Spectrometer (Thermo Fisher Scientific, Waltham, MA, USA) [33].

#### 4.3.2. Atomic Force Microscopy

Atomic force microscopy (AFM) was conducted to observe the surface irregularity of the prepared hydrogel CP2b and CP2b-GNS using Nano-Solver, NT-MDT, Moscow, Russia. The dried hydrogel samples were tested in ambient conditions whereby silicon nitride tip was used to scan the sample in semi-contact mode [29]. The area of the samples used for the analysis was 5 µm × 5 µm.

#### 4.3.3. Contact Angle Measurement

For the prepared hydrogel samples CP2b and CP2b-GNS, surface wettability examination was conducted using a contact angle measurement system (SEO-Phoenix-MT(A), SEO (Surface Electro Optics), Saneop-ro, Gwonseon-gu, Suwon-si, Gyeonggi-do, Republic of Korea)). Each sample measurement was recorded within a few seconds of placing the drop automatically. All the samples were tested in ambient conditions and at two different test surfaces [23].

### 4.4. Swelling Studies

We evaluated absorption capacity of the hydrogels (CS/PVA), (CS/PVA/GNS) in acid-base solutions, distilled water at various temperatures. After drying, the films were divided into tiny pieces and weighed. After that, the samples were allowed to swell in distilled water, acidic and basic solutions, at various temperatures (37° and 43°). At predefined (10 min and 20 min) intervals, samples were separated from the solutions, carefully cleaned using filter paper to get rid of water, and then weighed once more and calculated via formula. The swelling analysis was carried out three times, and the swelling analysis was derived from the average data.

(5) $$\mathrm{Degree}\;\mathrm{of}\;\mathrm{swelling}\;(\%)=\frac{M-Md}{Md}\times 100$$

where *Md* is the sample’s dry weight before to being submerged in the given solution, and *M* is the sample’s weight after its immersion in the given solution [41].

### 4.5. Degradation Analysis

The hydrogel sheets that had dried were divided into squares and accurately weighed (30 mg). PBS solution with a pH of 7.4 was used for the degradation analysis, and samples were allowed to incubate at 37 °C for 7 days. The examination of degradation was done thrice, with the average outcomes serving as the basis for the analysis [41].

The following equation was used to determine the degradation of hydrogels.

(6) $$\mathrm{Weight}\;\mathrm{loss}\;(\%)=\frac{W0-Wt}{W0}\times 100$$

where *Wt* is the hydrogel weight at time “*t*” and *W*0 is the hydrogel weight at beginning.

### 4.6. Loading and Release of Drug from Hydrogel

#### Drug Loading

Homogeneous CS/PVA/GNS solutions were produced as stated in the CS/PVA/GNS Solution Preparation Section. Then, with continuous stirring, 20 mg of Methotrexate (MTX) was incorporated into the aforementioned solutions for the production of a homogenous solution of CS/PVA/GNS/MTX. In the end, the solution of CS/PVA/GNS/MTX was incubated at 37 °C for 1 h to produce the MTX-loaded CS/PVA/GNS hydrogel [7].

### 4.7. In Vitro Release Test

The dry hydrogel containing drug was submerged in PBS (100 mL). With the help of a syringe, samples of hydrogels were taken after every 10 min for up to 6 h, and an equivalent amount of fresh PBS was replenished after each sampling. To analyze drug release behavior, the sampling method was extended to 420 min. The drug concentration in the sample was measured at 325 nm in a UV-visible spectrophotometer (Spectro-115U, Reference 3000, Gamry Instruments, Warminster, PA, USA) against a reference at the above-mentioned periodic intervals (PBS was used as a blank). A standard curve was used to determine the quantity of medication [23].

### 4.8. Hemolysis

Hemolysis provides information about the damage to the erythrocytic membranes (red blood cells). By monitoring the release of hemoglobin in the blood plasma, it can be located. The synthesized G2b-GNS hydrogel’s in vitro hemolytic potential is assessed by the hemolytic analysis. The saline solution and dry hydrogel were equilibrated for 24 h at 37 ± 0.5 °C. Then, 0.25 mL of human blood was applied to the wet hydrogel. Then 2.0 mL of saline solution was applied to the hydrogel’s surface after 20 min. The sample was then incubated for 60 min at 37 + 0.5 °C. 0.25 mL of human blood was mixed with 2 mL of sterile, distilled water to create a positive control. A negative control was implemented using the saline solution [42]. The samples that had been cultured were gathered to measure their optical densities at 545 nm. Hemolytic % was determined following the formula:

(7) $$\mathrm{Hemolysis}\;(\%)=\frac{\left(absorbance\;of\;test\;sample-absorbance\;of\;negative\;control\right)}{\left(absorbance\;of\;positive\;control-absorbance\;of\;negative\;control\right)}\times 100$$

### 4.9. Antibacterial Activity Evaluation

#### 4.9.1. Bacteria Culture

Using the well diffusion method, it was identified whether the hydrogels had antibacterial activity against Gram +ve (*S. aureus*), and Gram −ve (*E. coli*) bacteria that cause serious infections and diseases [40].

#### 4.9.2. Cell Culture

The growth medium used for the cells included 10% FBS, 20% penicillin-streptomycin, and DMEM. The cells were produced in an atmosphere with 5% CO_2_ at 37 °C. Prior to testing, HepG2 cells were cultivated logarithmically and allowed to reach 70% proliferation [39].

### 4.10. MTT Cell Viability Assay

The anti-cancer drug MTX was used to test whether various cell lines exhibited anti-proliferative characteristics utilizing the (MTT) assay. In the colorimetric MTT assay, living cells transform tetrazolium salt into formazan crystals. The cells were calculated using a hemocytometer in a cell culture flask. A 96-well plate was prepared by seeding 3.2 × 10^4^ cells into each well and adding 100 µL of medium. The 96-well plate was allowed to incubate at 37 °C, 5% CO_2_, and 95% humidity until 70–80% confluency was attained. The wells in the 96-well plate were cleaned using a 1X PBS solution.

Cells were subjected to a variety of stimuli by serial dilution, such as MTX-loaded CP2b-GNS GNS hydrogels and DMSO as a control. The cells were measured after a 24 h exposure to different conditions. Each well in the 96-well plate acquired 10 mL of MTT (5 g/mL) and 150 µL of solubilization buffer during incubation. To ensure total solubilization, the culture plate was gently spun and absorbance was taken at 570 nm using an ELISA plate reader [39].

### 4.11. Statistical Analysis

The acquired experimental data was calculated using statistical software (IBM, SPSS Statistics version 21, Chicago, IL, USA) and are shown as mean and standard errors (mean ± S.E). In figures, error bars represent the means and standard errors of mean (*p* < 0.05).

## Figures and Tables

**Table 1:** Surface roughness of the prepared hydrogels CP2b and CP2b-GNS.

Sample Name	Average Roughness (nm)	Root Mean Square Roughness (nm)
CP2b	7.469 nm	10.694 nm
CP2b-GNS	2.803 nm	3.552 nm

**Table 2:** Drug release kinetics by different models.

Models	Intercept	Slope	R^2^
Zero Order	2.9311	0.0119	0.9369
First Order	0.4926	0.0011	0.8927
Higuchi model	39.281	6.5261	0.9312
Korsmeyer-Peppas model	0.0602	0.3491	0.9659

**Table 3:** Formulations used for the preparation of Chitosan/PVA hydrogels.

Formulations	CS	PVA	Cross Linker (TEOS)
CP1	0.2 g	0.8 g	--
CP2	0.4 g	0.6 g	--
CP3	0.6 g	0.4 g	--
CP2a	0.6 g	0.4 g	50 µL
CP2b	0.6 g	0.4 g	100 µL
CP2c	0.6 g	0.4 g	150 µL

**Table 4:** Formulation used for the preparation of GNS based Chitosan/PVA hydrogel.

Formulations	CS	PVA	GNS	TEOS
CP2b-GNS	0.6 g	0.4 g	0.5%	100 µL

## Data Availability

The data presented in this study are openly available in article.
